# Users remain overlooked: Shared decision‐making processes for people with anxiety disorders

**DOI:** 10.1111/jpm.13095

**Published:** 2024-08-20

**Authors:** Amelia Villena, María M. Hurtado, Clara Gómez, Gisela Amor, Amanda Vega, José Miguel Morales‐Asencio

**Affiliations:** ^1^ Mental Health Unit Regional University Hospital of Málaga Málaga Spain; ^2^ Faculty of Health Sciences Universidad de Málaga Málaga Spain; ^3^ Instituto de Investigación Biomédica de Málaga (IBIMA) Universidad de Málaga Málaga Spain; ^4^ Badalona Care Services Barcelona Spain

**Keywords:** anxiety, coping, patient‐centred care, patient participation, shared decision‐making

## Abstract

**What Is Known on the Subject?:**

- Patients do not always receive enough information about their diagnosis and their perceived participation in decision‐making about their treatment is low.
- Some participants reported feeling very uncertain when the physician invited them to choose between these options. Others users expressed their satisfaction with the trend away from paternalistic attitudes in the health system.
- There is a trend towards pharmacological prescription as a first approximation. This contrasts with the recommendations of scientific organizations based on evidence and cost‐effectiveness studies on the offer of psychological interventions as the first option.
- The user groups pointed out that active coping, based on exposure to anxiety‐generating situations, made a significant contribution to alleviating their anxiety disorders. However, some of those interviewed rejected this type of intervention.

**What the Paper Adds to Existing Knowledge?:**

- Users diagnosed with anxiety disorders miss more information about the disorder and participation in its treatment.
- Opposite positions coexist in terms of participation in the choice of treatment.
- Pharmacological treatment is most commonly the first option offered.

**What Are the Implications for Practice?:**

- This study is an example in itself of the involvement of users in the healthcare process, and therefore placing them at the centre of attention, as reflected in healthcare policies and clinical practice guidelines.
- It promotes the identification of needs that users diagnosed of anxiety disorders may have, with the aim of putting in place, from healthcare professionals and health services, the necessary supports adapted to these.
- Mental health nurses are well‐positioned to offer support and guidance during the process of involvement and shared decision‐making.

**Abstract:**

**Introduction:**

An essential aspect of mental health treatment and recovery is the degree of involvement by health service users in the process.

**Aim/Question:**

Explore the values, demands and preferences of persons diagnosed with anxiety disorders, their participation in the treatment provided, and the response of the health system in this regard.

**Methods:**

A qualitative study was conducted, with 51 participants. Nine focus groups and four in‐depth interviews took place.

**Results:**

Three broad categories were identified: (1) diagnosis; (2) treatment options offered and shared decision‐making; and (3) coping with the disorder. Sometimes patients do not receive enough information to cover their needs. A trend towards drug prescription as a first approach was observed, while active coping based on exposure to anxiogenic situations was indicated as the most effective option.

**Conclusion:**

Shared decision‐making is a necessary aspect of treatment, and the therapeutic process should be adapted to match the service user's preferences, values and needs.

**Implications for the Practice:**

This research identifies the needs of patients diagnosed with anxiety disorders and promotes, therefore, from healthcare professionals and services, the provision of measures to meet these needs.

## 1 INTRODUCTION

From the perspective of the mental health recovery model, an approach to mental health treatment is proposed focused on the goals, values, motivations and preferences of users, in which a higher priority is assigned to these aspects than to achieving a total absence of symptoms (Davidson & Roe, 2007; Shepherd et al., 2008). Under this premise, one of the requisites for recovery is that of participation by service users in decision‐making about their treatment and in the organization of this process. Although this greater user involvement in planning and developing health services, as well as in health research, is not exclusive to the field of mental health, it does constitute a fundamental part of the mental health recovery model (Davidson & Roe, 2007; Matthias et al., 2012; Shepherd et al., 2008). Several models of user involvement have been deployed, such as patient‐centred care, shared decision‐making, or patient participation, although the recovery model goes beyond since puts emphasis on health, strengths and wellness. Thus, users and their families are considered as active participants in designing care systems (Storm & Edwards, 2013).

Clinical Practice Guidelines are an important area where patient engagement contribute to empowering users in well‐informed healthcare decisions and respecting the rights of citizens in healthcare policies and practices; international guideline standards have stated users involvement in guidelines development as a key element of high‐quality evidence‐based guidance (Armstrong et al., 2017). NICE (National Institute for Health and Clinical Excellence) and SIGN (Scottish Intercollegiate Guidelines Network), are two of the most relevant organizations internationally in the development of guidelines that have incorporated this approach. They do so by means of a ‘person‐centred approach’ (Department of Health, 2010, 2012; Drake et al., 2010; McCormack et al., 2010; McCormack & McCance, 2016), which results in enhanced service development, improved staff attitudes and increased self‐esteem, satisfaction and empowerment among users (Crawford et al., 2002; Tambuyzer & Van Audenhove, 2015).

In Spain, both professional and citizen organizations encourage user participation as a real and effective involvement in decision‐making at all levels of the social and health system, public or private, of the people who use it (Confederación Española de Agrupaciones de Familiares y Personas con Enfermedad Mental, 2014).

Anxiety disorder is the most frequent diagnosis made in the field of mental health, and an estimated 25% of the population will have a diagnosis of this type during their lives (Duncan et al., 2010; Edwards et al., 2023; García‐Herrera Pérez‐Bryan et al., 2015; Goossensen et al., 2007; Goss et al., 2008; Liebherz et al., 2015; McCabe et al., 2013; Remes et al., 2016; Rodenburg‐Vandenbussche et al., 2020; Simmons et al., 2011; Val & Míguez, 2023; Zhang et al., 2022). Despite this prevalence, there is a shortage of studies on the involvement and participation in decision‐making among patients with anxiety, as highlighted by numerous authors (Aoki et al., 2022; Burns et al., 2021; Marshall et al., 2021; Ramos‐García et al., 2021). Specifically, in the Spanish context, where a public universal coverage health care systems exists, no previous studies have focused on this issue. The limited studies available often focus on specific types of diagnoses or include patients with other diagnoses, such as depressive disorders and other types of disorders (De Las Cuevas & Peñate, 2016; Mundal et al., 2021; Ramos‐García et al., 2021) or in health care systems without universal coverage. There seems to be a preference among patients to actively participate in decisions related to their disorder (Aoki et al., 2022; Ramos‐García et al., 2021; Rodenburg‐Vandenbussche et al., 2020; Schladitz et al., 2023). Moreover, personalized care planning and shared decision making are the least frequent ingredients of collaborative mental health care in depression and anxiety programs (Menear et al., 2020). In the qualitative study by Rodenburg‐Vandenbussche et al. (2020), which included patients with anxiety disorders and obsessive‐compulsive disorder, they believed that Shared Decision‐Making (SDM) should be common practice. This perspective was grounded in the autonomy they held over their own bodies, and they felt a sense of responsibility for their treatments. Liebherz et al. (2015) reported that most patients with anxiety disorders had a high level of information and decision‐making needs, especially regarding the initiation of psychotherapy and the type of treatment to be received. Ramos‐García et al. (2021), in a study conducted with individuals diagnosed with generalized anxiety disorder, also found that the majority preferred an active or collaborative role in decision‐making, but 53% did not perceive it that way. Additionally, users expressed a desire for more information about their disorder, self‐help groups, and therapeutic options. Therefore, research on collaborative care programs within the context of providing care for individuals with anxiety disorders indicates that shared decision‐making is not the norm (Menear et al., 2022).

Furthermore, user involvement is related to greater adherence to treatment, increased satisfaction (Loh et al., 2007) and an alleviation in depressive symptoms (Clever et al., 2006). Similar conclusions have been drawn by studies focusing on patients with anxiety (Liebherz et al., 2015; Patel & Bakken, 2010). However, the systematic review by Aoki et al. (2022) highlights a low level of certainty regarding the effects of shared decision‐making on users with mental disorders, suggesting the need for further research. There is also a scarcity of studies on this topic specifically among patients with anxiety disorders, or they have been carried out with quantitative methods (Aoki et al., 2022; Marshall et al., 2021; Menear et al., 2022; Ramos‐García et al., 2021; Tlach et al., 2015).

Although anxiety disorders are not the only disorders where such involvement is relevant, several important issues have been raised about user involvement in treatment choice in the case of persons with anxiety disorders. On the one hand, as there are numerous psychological therapies and pharmacological treatments with similar levels of efficacy, it has been argued that users should be able to determine the treatment of choice according to their values and preferences. In fact, it has been observed that for some situations, such as generalized anxiety disorder, the adoption of the user's preferred psychological therapy could improve outcomes (Levy Berg et al., 2008). Moreover, the psychological therapies for which the evidence of efficacy is strongest (such as behavioural therapy and cognitive behavioural therapy) actually require participants to play an active role in performing the tasks agreed upon during therapy sessions. Lastly, according to the stepped‐care model developed by NICE and followed in several countries, including Spain (García‐Herrera Pérez‐Bryan et al., 2011, 2015), the users play an active role both in the choice of treatment (when several equivalents are available) and in the therapeutic process itself, especially when the activities involved are of low intensity, such as self‐help guidance. Lastly, difficulties in the continuity of treatments in common mental disorders could also be solved by greater participation of the user in decision‐making (Chong et al., 2011; Vergouwen et al., 2003).

In this context, the present study focuses on the values, demands and preferences of patients diagnosed with different anxiety disorders, and considers the care received in this respect in a public health system with universal coverage. Users' perceptions about their role in the therapeutic process, their understanding of what is happening to them, their search for recovery strategies, and the role of the health care system in this regard are also explored.

## 2 METHODS

### 2.1 Context of study and sample

A qualitative study was carried out in two community mental health units, one in an urban area and the other in a rural area. The first of the units owned at Service of Mental Health of the Regional University Hospital of Malaga (Spain) and it corresponds to an urban area in the city of Malaga. This unit usually serves a population of 165,000 inhabitants. The second unit owned al Health Management Area of Northern Cordoba (Spain) and it corresponds to a rural area in the city of Pozoblanco. This unit assists 85,206 people annually.

The study was conducted from a critical discourse perspective (Hodges et al., 2008). Its aim was to broaden understanding of the problem beyond their individual perspective, to also encompass their view of the institutional practices that they experienced when received health care for their anxiety disorder, in the context of a public health care system with universal coverage. Public mental health care tend to have increased waiting periods for access, and frequent resource limitation to offer continuity and prolonged therapies. Conversely, public healthcare is provided at no cost to the patient. These features may influence on users perception and preferences on their involvement in share decision‐making (Mundal et al., 2021).

By a purposive sampling considering the criteria listed in Table 1, the full list of users diagnosed with social anxiety disorder (SAD), obsessive‐compulsive disorder (OCD), anxiety generalized disorder (GAD) and panic disorder was revised. Although, for some years now, OCD has not been considered by classification systems as an anxiety disorder (APA, 2014; WHO, 2022), it was included due to presenting a functional and contextual analysis similar to that of a phobic anxiety disorder (Hurtado et al., 2023). All those selected were telephoned to explain the objectives of the study and to request their participation. Those who were subsequently interviewed were again informed about the study goals, assured that all data would be treated in strict confidence and asked to sign the informed consent form. The sample size was determined in accordance with the principle of data saturation during data collection and analysis. Saturation was evaluated every two interviews by analysing the codes and categories and performing triangulation. Based on the results, the need for new interviews was decided.

**TABLE 1:** Inclusion and exclusion criteria for the selection of participants.

**Inclusion criteria**
Having a diagnosis of social anxiety disorder, obsessive‐compulsive disorder, anxiety generalized disorder or panic disorder by psychiatrists or clinical psychologists according the DSM 5. Having at least two appointments in Mental Health, in addition to consultations in PC, to ensure that they had enough experience with different health care providers. *Users diagnosed with post‐traumatic stress disorder are excluded, as recent studies warn that investigating individual or group traumatic events and related factors may lead to an aggravation of symptoms (American Psychiatric Association, 2013).
**Exclusion criteria**
Under age of 18. Having a diagnosis of a serious mental disorder (psychotic or bipolar disorder) or a comorbid addiction (alcohol or illegal drugs). Having mental retardation, limited intelligence quotient, significant functional impairment or a marginal social status that prevented their proper monitoring (homeless, in prison…). Abandoned follow‐up without having being discharged by a health professional.

### 2.2 Analyses

The study data were compiled from in‐depth semi‐structured individual interviews with users suffering social anxiety disorder, and from focus group interviews with participants presenting any other type of anxiety diagnosis. The focus group is chosen as the primary method of data collection because it allows access to opinions, experiences and perceptions that would be less accessible without the interaction of group members (Onocko‐Campos et al., 2017). In the case of users with social phobia, individual interviews were conducted so that the symptoms of the disorder did not interfere with their participation. In the focus groups, the diagnosis was the only segregation variable used. The topics proposed to the participants were determined by the researchers on the basis of a prior literature review and by consensus (see Appendix 1). The following topics were discussed: the assessment of the professionals involved (in Primary Care and Mental Health); the types of approaches offered in the public health system (including psychological and pharmacological options) and their perceived usefulness; and personal resources for coping with mental disorder. The interviews, which were flexible and lasted 90–120 min, were carried out by a neutral interviewer (not a member of the research team), who was highly experienced in conducting qualitative interviews. Moreover, an observer took some notes on the situation of each participant and the non‐verbal aspects that could help to understand the interactions between the participants. Interviews took place in a training room in a health centre other than the one in which they received the treatment.

All interviews were recorded and the audiotapes were transcribed verbatim, after which a content analysis was performed according to the principles suggested by Taylor et al., 2015. The transcripts were read to identify the main emerging themes, which were subsequently coded by a member of the research team. The members of the research team involved in the analysis, two were specialists in clinical psychology, and the third was undergoing specialized healthcare training, as well as having specific training and experience in qualitative methodology. These codes were then triangulated, via reviews by two other members of the research team. Any differences in the codes proposed were discussed and resolved among the researchers. The codes were grouped into categories and subcategories, and analysed taking into account the potential influence of the researchers. All analyses were performed using ATLAS.ti (version 7, Berlin, Germany) software for qualitative data analysis.

To ensure the credibility and validity of the results obtained, the criteria of credibility, transferability, consistency and confirmability, as identified by Guba and Lincoln in this respect (Guba & Lincoln, 2000), were taken into account. To ensure the credibility of the analysis process, we proceeded to the triangulation of codes and categories. Transferability was strengthened by the completeness of data collection in each group, across multiple potential situations, scenarios and experiences with anxiety disorders. The criteria of consistency and reproducibility of the data were achieved by a detailed and documented analysis process strategy and the context in which data collection took place. From the point of view of confirmability and reflexivity before the start of the study, the researchers conducted an analysis of their own preconceptions and expectations regarding the study results to compare to what extent could subsequently influence over process. Additionally, a moderator/interviewer neutral (not belonging to the research team) and experienced in qualitative interviews was used.

## 3 RESULTS

The study sample was composed of 51 patients, 35 women and 16 men, with a mean age of 46.68 years. A detailed description of the participants is included in Table 2. Nine focus group sessions were held: two with patients diagnosed with OCD, three with patients with panic disorder and four with patients who had been diagnosed with GAD. In addition, four in‐depth interviews were conducted with patients diagnosed with SAD.

**TABLE 2:** Characteristics of the participants.

Primary diagnosis	*n*	Age	Gender	
			Women	Men
GAD	24	53.12	18	6
OCD	12	43.16	7	5
Panic Disorder	11	43.27	8	3
SAD	4	28	2	2
Total	51	46.68	35	16

Analysis of the content of these sessions enabled three thematic categories to be identified: (1) Diagnosis; (2) Treatment options offered and shared decision‐making process; (3) Coping with the disorder: active versus passive coping.

### 3.1 Diagnosis

The first step for users to be able to participate effectively in the therapeutic process is for them to realize what is happening to them, via the diagnosis made. Some anxiety disorders are diagnosed more quickly than others; sometimes during the first consultation, as can be the case with OCD, and at other times, only gradually, probably based on the user's ability to assimilate it.

Before, he always told me I was obsessive, but he didn't put a name on it, which I think has been positive for me since maybe if they had told me ‘Look, you have such and such…’, maybe that would have worried me more. (Man, 23 years old, diagnosed with OCD)

In some cases, too, users were told nothing at the beginning of their care, or were given confusing information.

These episodes repeated very often, and every time I went to the emergency room, they told me ‘You have to visit a psychologist’. And I said ‘What do you mean, a psychologist, I'm strong, I can handle this’, and much more besides. But nobody told me it was anxiety. Because I thought anxiety was a state of nerves. I didn't know that anxiety was so deep and so bad. It was completely unknown. (Woman, 63 years old, diagnosed with GAD)

However, with the other disorders, participants most commonly referred to anxiety or ‘nerves’, in general, and had been offered no specific diagnosis or explanation. It is noteworthy that, except for those diagnosed with OCD, hardly any of the participants mentioned a specific diagnosis.

Information regarding the characteristics of the disorder, the nature of anxiety, the course of the condition and general guidance on management (known as psychoeducation) is infrequent at first, usually being offered at later stages of healthcare, when the patient is already receiving specialized treatment. This intervention fulfils two functions: it reassures patients and motivates them to take an active approach to coping with their disorder.

The scientific explanation in a structured way of the things I felt was particularly helpful to me. It had seemed to me that I was making them up, and the moment a professional talked about it properly, it was as if he was giving you permission, as if he was backing you up and making you relaxed. He said, ‘Well, it's not something you're inventing, it happens, it's well known and you're not making it up’. For me, after that, it was like before and after. (Woman, 42 years old, diagnosed with panic disorder)

The lack of information causes many users to use the internet to find out more about their problem, especially in the case of OCD. The perceived usefulness of these experiences was varied. Some people found this search detrimental, perhaps because it heightened their tendency to worry. On the other hand, some persons used the internet to establish online support groups and found the experience very positive.

My experience on the internet, since 2010, we created a group of people from all over Spain who have obsessive disorders, and there we talk, and everything, and someone who has already been discharged and is cured may be the leader of that group, and be helping others. (Woman, 33 years old, diagnosed with OCD)

Also, if you start looking for the disorder, it's like you focus your attention on the problem that you have… Our problem is solved precisely by not paying attention to it. (Woman, 43 years old, diagnosed with OCD)

### 3.2 Treatment options offered and shared decision making

According to this user group, there seem to be two relational styles from health care staff: a paternalistic one, and increasingly another that is more open, in which decisions are shared. However, the latter trend still generates uncertainty in some users.

It's true that the dynamics of doctors have changed, towards offering rather than imposing. This has its pros and its cons. The professional is really the one who knows best, I think, and if he asks you… ‘Well, what do I know? Well, as you say, that's why I came here’. To some extent, this may be a way of safeguarding himself, anyway, or maybe not. I don't know. But, as I said, on the one hand I'm in favour, and on the other it isn't that I think it's bad, but it generates a little uncertainty when you yourself have to decide what the treatment should be. (Man, 50 years old, diagnosed with OCD)

The doctor, the psychiatrist, recommended that I also have therapy with a psychologist and I agreed, I said to her ‘you are the boss’.(Man, 42 years old diagnosed with OCD)

However, all users agreed that their opinions were taken into account regarding the maintenance, change or withdrawal of pharmacological treatment during subsequent consultations.

After a month, I said, ‘The pill isn't doing any good’, and she said, ‘Well, then, let's try this one’. (Man, 42 years old, diagnosed with OCD)

According to this user group, the psychotherapy offered varied according to the type of disorder diagnosed. For example, psychological intervention was often offered to patients with social anxiety from the first consultation, either as a single intervention or together with pharmacological treatment. Similarly, persons with panic disorder were sometimes offered psychological intervention, via group psychotherapy, from the outset.

I explained to the psychiatrist what was happening to me. She asked me if I was taking medication and I said no, and I didn't want to take anything, either. So, straight away, she referred me to the mental health unit, to see the psychologist. I spoke to her a couple of times, and when I explained the problem she gave me another session, and then she told me about the therapy group. (Woman, 23 years old, diagnosed with panic disorder)

In the case of the panic disorder therapy group, some users even proactively requested this treatment, when they heard from other users how it worked for them. As regards other mental health disorders, pharmacological treatment is usually offered first and only later – and then only in some cases—a psychological intervention may be suggested.

The psychiatrist later told me about cognitive behavioural therapy with a psychologist, but he said that I should first take the medication until I was a little better. Then, it was me who asked for it, I asked him to refer me to the psychologist. (Woman, 44 years old, diagnosed with OCD)

In general, there is a preference for psychological interventions. This, together with the fact that it is not always offered, prompted comments that this option was something that had been missing from the health care provided.

I think that, if from the beginning they had given me therapy, instead of medication, and they had approached it in another way, I think that things would have gone differently, I think, in my case, if they had taught me to de‐dramatise, if they had explained things to me differently. (Woman, 43 years old, diagnosed with OCD)

Another form of proactive participation is the creation of support groups coordinated via new technologies by the patients themselves, as a therapy group. This innovation was mentioned by three of the participants.

About two or three years ago, I was in another group helping with anxiety issues and it was great, too. In fact, with the people from that group, we still keep in touch by WhatsApp and we call ourselves ‘the therapists’ and everything's very good, we're very happy. (Woman, 39 years old, diagnosed with GAD)

### 3.3 Coping with the disorder: Active versus passive strategies

Regarding the possibility of actively participating in the recovery process by changing their habits and by adopting coping strategies, the vast majority of users agreed this was totally necessary.

You don't feel the changes are because of the pills, but because of what you're doing yourself. (Woman, 19 years old, diagnosed with social anxiety disorder).

There the active part is you and, if you do not put that part, neither medication nor the best professional in the world … There is a part that is you… (Woman, 44 years old, diagnosed with OCD)

Therapy is often used to guide users in this task. Before, the patient only knew that something had to be done, but with therapy they feel that someone is showing the way and they can begin to take steps in another direction.

Therapy, talking and, well, doing the things that I don't normally dare to do. (Man, 27 years old, diagnosed with social anxiety disorder)

The user groups referred to three main strategies to combat their symptoms. The first was to stay physically and/or mentally active.

What I've realised is that you've got to have something to think about. If you like fishing, well, go fishing. If you like … what you like is what you have to look for. (Man, 57 years old, diagnosed with GAD)

Many users, especially those with panic disorder, also referred to breathing and relaxation techniques for managing an initial anxiety crisis.

Breathing. Fundamentally, it's breathing. For me, it's taking a good breath. Other times, you can't breathe very fast because you get dizzy, but as the psychologist taught me, ‘Count to four, breathe in, counting to four, and then breathe out, counting to four. Keep on doing that and you'll see how the pressure in your chest is relieved’. I did it, and it worked. So, if I get uncomfortable I start to breathe and it passes. If I can control it with my breathing, obviously there's nothing wrong with my heart. (Man, 62 years old, diagnosed with panic disorder)

However, the strategy most frequently cited by this group was that of exposure to anxiogenic situations. By exposing themselves, little by little, to situations that might provoke anxiety attacks, these users came to realize that the terrible outcome they had feared would not actually happen. This experience helped them greatly in future situations of this kind. In fact, those who used this type of strategy most were also those who reported the best recovery from their problem.

The psychologist really tells you clearly. He says, ‘You must do this and this’. You don't want to do it because it means changing how you do things and you don't want anyone to touch you because you're doing it that way. So, you go out, feeling angry, you go out saying, ‘The hell with it all’. But hey, after the same experience happens again and you feel bad, if you follow that advice, that therapy the psychologist suggested, and you see that you're improving… Then you realise it helps you face down the fear… (Man, 23 years old, diagnosed with OCD)

To put these strategies into effect, especially those such as exposure techniques that involve emotional discomfort, users must learn to use covert self‐control techniques, such as avoiding negative thinking, self‐reinforcement or recalling the explanations received about their symptoms, instead of letting catastrophic thoughts take over.

When I had a panic or anxiety attack, my heart used to start racing. I thought I'd never get over it. I also thought I was going crazy. Lots of thoughts. So, of course, I didn't know what it was. The answer was in the techniques they taught me. They told me I wasn't going to die, things that you think because you don't know what's happening. They also taught me to control my breathing. And to face up to the situations that make me panic. (Woman, 26 years old, diagnosed with panic disorder)

On the other hand, some users were unable to use active coping strategies, often because they sought to avoid any situation that could provoke intense emotional discomfort. For this subgroup, the evolution of their condition was perceived to be independent of their behaviour.

I have a four‐year‐old niece who wants me to come out with her. No way. I don't want to. They call me through the window, but I don't open the window, I keep it closed, so that I'm in the dark all day. So, I keep my little window closed, and then I say ‘Oh, I didn't hear you because of the TV’. (Woman, 51 years old, diagnosed with GAD)

I do not suffer from anxiety attacks but it is because I do not go to the places that induce those attacks. (Woman, 63 years old, diagnosed with panic disorder)

## 4 DISCUSSION

### 4.1 Summary of findings and implications for the clinical practice

Our study results show that the participation mechanisms provided by the health care system for patients diagnosed with various anxiety disorders are, in general, inadequate. The different mechanisms employed by these patients to cope with their condition are also highlights in this study. This study represents an approach to the needs of patients with different anxiety disorders regarding their involvement and decision‐making in the healthcare process, addressing the scarcity of studies on this matter.

To facilitate decision‐making and active involvement in the process, patients must receive sufficient information about what is happening to them and about the treatment options available. However, according to the user groups consulted in this study, patients do not always receive enough information, in areas such as the identification of their disorder and the diagnosis made. In consequence, it is difficult for these users of the health service to participate effectively in decision‐making regarding their treatment. This finding has been also reported in prior studies in users with generalized anxiety (Ramos‐García et al., 2021).

Due to the lack of information received about the diagnosis and about the therapeutic options available, some participants reported feeling very uncertain when the physician invited them to choose between these options. In fact, it has been observed that some patients with emotional disorders prefer the health professional to decide such questions (Joosten et al., 2008; Mundal et al., 2021). Some patient‐related characteristics, such as gender, health anxiety and employment status, are known to be associated with their satisfaction in relation to shared decision‐making (Bot et al., 2014; Mundal et al., 2021). In the present study, the most of user groups expressed their satisfaction with the trend away from paternalistic attitudes in the health system, which corroborates previous research findings in this respect (Chewning et al., 2012; De Las Cuevas & Peñate, 2016; Joosten et al., 2008; Mundal et al., 2021; Rodenburg‐Vandenbussche et al., 2020).

Mental health nurses, as part of the multidisciplinary care team, frequently interact with individuals receiving mental health services and are well‐positioned to offer support and guidance during the process of involvement and shared decision‐making (Gurtner et al., 2021), as it has been reported in other areas of mental health care (Song & Song, 2023).

In contrast to recommendations based on prior empirical evidence on psychotherapeutic options for patients with anxiety disorders (García‐Herrera Pérez‐Bryan et al., 2015), our analysis revealed a trend towards pharmacological prescription as the first approach. Therefore, a gap exists between the practices reported by these patients and the recommendations of evidence‐based scientific organizations, together with numerous long‐term cost‐effectiveness studies of this question (Mavranezouli et al., 2015; National Collaborating Centre for Mental Health, 2006, 2013; National Institute for Health and Care Excellence: Clinical Guidelines, 2011; Tolin et al., 2011; van Apeldoorn et al., 2014).

The user groups pointed out that active coping, based on exposure to anxiety‐generating situations, made a significant contribution to alleviating their anxiety disorders (Barlow et al., 2000; Foa et al., 2005; Hoyer et al., 2009; Rapee et al., 2009). However, some of those interviewed rejected this type of intervention and studies have shown that many patients refuse to follow this type of therapeutic indication (American Psychiatric Association, 2013; Flückiger et al., 2016; Haug et al., 2016; Johnson et al., 2014). In addition to coping strategies, the existence of groups naturally generated by participation in group therapeutic activities was found to be useful by many patients. In a study by Ramos‐García et al., more than 65% of interviewed patients with generalized anxiety disorder expressed a lack of information regarding the existence of self‐help groups (Ramos‐García et al., 2021). Other therapeutic factors that have traditionally been found useful are universality, vicarious learning and mutual aid arising from group therapy (Behenck, Gomes, & Heldt, 2016; Behenck, Wesner, et al., 2016; Vinogradov & Yalom, 1996).

In this regard, too, an emerging theme was detected in the focus groups regarding the perceived value of online support groups. With the expanding use of social networks, many internet support groups for persons with mental health issues have been established (Callan et al., 2017; Mohr et al., 2013; Zabinski et al., 2003). Although their impact or otherwise in achieving any remission in symptoms is not yet clear (Callan et al., 2017; Geramita et al., 2018; Griffiths et al., 2012; Rollman et al., 2018), participation in these groups is associated with other advantages, such as access to practical information, encouraging hope and offering round‐the‐clock availability (Castelnuovo et al., 2003). On the other hand, active counselling and guidance on identifying reliable sources of information is very necessary, because patients are frequently unable to verify the reliability of medical information supplied on the internet (Baup & Verdoux, 2017; Ramos‐García et al., 2021).

In the study, some differences among the subgroups of disorders are found. On one hand, a diagnosis is delivered earlier in the case of individuals with OCD than in those with other anxiety disorders. The specificity and clarity of OCD symptoms, compared to the more diffuse and general symptoms of other anxiety disorders such as generalized anxiety disorder, facilitate its detection (APA, 2014). On the other hand, another difference is that for individuals with social phobia and panic disorder, psychological intervention is offered at the beginning of care. This difference may be due, on one hand, to the existence of a specific integrated care process for these disorders, which has led to the establishment of therapeutic programs in our context (Diaz del Peral, 2011), and, on the other hand, to the demonstrated effectiveness of these interventions in these cases compared to pharmacological approaches (National Collaborating Centre for Mental Health, 2013; National Institute for Health and Care Excellence: Clinical Guidelines, 2011).

The results of the present study demonstrate that many individuals diagnosed with anxiety disorder are inadequately involved in the decision‐making process regarding their condition, lack information on how to cope, and doubt the usefulness of the treatment received. Therefore, it is necessary for both healthcare professionals and managing bodies to review the clinical practices applied to these patients and to advocate for adjusting them to the person‐centred approach on which clinical guidelines are based. This adjustment will ensure the respect of citizens' needs and preferences in healthcare policies and practices. Finally, as an emerging theme, the use of online support groups for the management of anxiety problems by users has been identified, which deserves to be studied in greater depth in the future.

### 4.2 Limitations

This study has some limitations. First, a limitation of this study it could be a possible self‐selection bias in the users who agreed to participate in the focus groups, so that those subjects who rejected to participate could have different illness and health care experiences. This decision could be influenced by the severity level of their symptoms when they were invited to participate, as well as the satisfaction level with the healthcare received. To minimize this bias, both the people who made the recruitment calls and those who were present during the interviews were independent of the healthcare team. Additionally, the recruitment team presented participation in the study as an opportunity to highlight the deficiencies observed in the system and the healthcare response. Second, the interview transcripts were not returned to the participants for correction of possible transcription and interpretation errors. However, during the interviews, the interviewer provided summaries and syntheses of participants' statements, allowing for the correction of interpretation errors. Third, participants did not receive feedback on the results. Nevertheless, it was never denied that participants could have access to the results, upon request. From the perspective of result generalization, it is conceivable that these findings may vary depending on the care context encountered by other patients during the management of their anxiety disorder. Nevertheless, the interaction of paternalistic models in shared decision‐making is documented across multiple clinical settings (Menear et al., 2022).

## 5 RELEVANCE STATEMENT

Patients with anxiety disorders often require the care of mental health nurses. This study is an approach to the experience of these users with coping with the disorder and the care received from healthcare services. Identifying needs and preferences allows healthcare to address them and, therefore, places the user at the centre of healthcare, as promoted by health policies, clinical practice guidelines and the user movement.

On the other hand, psychoeducation about anxiety is a crucial therapeutic objective in addressing anxiety issues, and it is particularly relevant to implement it in the early stages of patient care. This goal constitutes an essential task for nursing professionals in both primary and specialized care.

## FUNDING INFORMATION

Open access publishing facilitated by the University of Málaga.

## ETHICS STATEMENT

The study was approved by the Ethics and Research Committee of Malaga (Spain). All methods were performed in accordance with the Declaration of Helsinki. Informed consent was obtained from all participants.

## Data Availability

The dataset supporting the conclusions of this article is available in the *Repositorio Institucional de la Universidad de Málaga* (RIUMA), in https://doi.org/10.24310/riuma.22705.

## APPENDIX 1 GUIDE USED IN FOCAL GROUPS AND INDIVIDUAL INTERVIEWS

### 1.1

When were you diagnosed and how old were you?

When did you seek help from the public health system and who did you contact? (Describe this first contact, please). What help did you have to access the health services? If you did not seek help personally, explain how you managed to access the services.

What possible treatments did they propose to you at the beginning? Did you reach an agreement with the healthcare provider on how to deal with the disorder?

What treatment did you receive? Describe the drug treatment and psychological therapies provided.

Was the treatment helpful? Describe what worked and what didn't.

Did you attend a support or therapy group? Was it helpful?

Did you require any hospital admission? Describe what it was like, both the positive and the negative aspects.

How would you describe your relationship with healthcare providers (family doctor, psychiatrist, psychologist, nurse, etc.)?

Did your family and friends help and support you?

In addition to the public health system, are you receiving health care elsewhere to help you with psychotic disorder, for example, private treatment? If so, describe what worked and what didn't for you.

How has the disorder changed over time?

How do you feel now?

How does psychosis affect your daily life (studies, work, relationships) and the lives of those close to you?

What strategies do you currently use to cope with the difficulties generated by the disorder?

## References

[jpm13095-bib-0001] American Psychiatric Association . (2013). Diagnostic and statistical manual of mental disorders (5th ed.). American Psychiatric Association.

[jpm13095-bib-0002] Aoki, Y. , Yaju, Y. , Utsumi, T. , Sanyaolu, L. , Storm, M. , Takaesu, Y. , Watanabe, K. , Watanabe, N. , Duncan, E. , & Edwards, A. G. (2022). Shared decision‐making interventions for people with mental health conditions. Cochrane Database of Systematic Reviews, 2022(11), CD007297. 10.1002/14651858.CD007297.pub3 PMC9650912 36367232

[jpm13095-bib-0003] APA . (2014). Diagnostic and statistical manual of mental disorders, fifth edition (DSM‐5). Editorial Médica Panamericada.

[jpm13095-bib-0004] Armstrong, M. J. , Rueda, J. , Gronseth, G. S. , & Mullins, C. D. (2017). Framework for enhancing clinical practice guidelines through continuous patient engagement. Health Expectations, 20(1), 3–10. 10.1111/hex.12467 27115476 PMC5217879

[jpm13095-bib-0005] Barlow, D. H. , Gorman, J. M. , Shear, M. K. , & Woods, S. W. (2000). Cognitive‐behavioral therapy, imipramine, or their combination for panic disorder: A randomized controlled trial. The Journal of the American Medical Association, 283(19), 2529–2536. 10.1001/jama.283.19.2529 10815116

[jpm13095-bib-0006] Baup, H. , & Verdoux, H. (2017). Frequency and pattern of internet use in patients with schizophrenia or bipolar disorders seeking medical information. Psychiatric Research, 247, 152–154. 10.1016/j.psychres.2016.11.028 27893996

[jpm13095-bib-0007] Behenck, A. , Gomes, J. B. , & Heldt, E. (2016). Patient rating of therapeutic factors and response to cognitive‐behavioral group therapy in patients with obsessive‐compulsive disorder. Issues in Mental Health Nursing, 37(6), 392–399. 10.3109/01612840.2016.1158335 27105227

[jpm13095-bib-0008] Behenck, A. , Wesner, C. , Finkler, D. , & Heldt, E. (2016). Contribution of group therapeutic factors to the outcome of cognitive‐behavioral therapy for patients with panic disorder. Archives of Psychiatric Nursing, 31, 142–146. 10.1016/j.apnu.2016.09.001 28359425

[jpm13095-bib-0009] Bot, A. G. , Bossen, J. K. , Herndon, J. H. , Ruchelsman, D. E. , Ring, D. , & Vranceanu, A. M. (2014). Informed shared decision‐making and patient satisfaction. Psychosomatics, 55(6), 586–594. 10.1016/j.psym.2013.12.013 24836165

[jpm13095-bib-0010] Burns, L. , Da Silva, A. L. , & John, A. (2021). Shared decision‐making preferences in mental health: Does age matter? A systematic review. Journal of Mental Health, 30(5), 634–645. 10.1080/09638237.2020.1793124 32662713

[jpm13095-bib-0011] Callan, J. A. , Wright, J. , Siegle, G. J. , Howland, R. H. , & Kepler, B. B. (2017). Use of computer and Mobile Technologies in the Treatment of depression. Archives of Psychiatric Nursing, 31(3), 311–318. 10.1016/j.apnu.2016.10.002 28499574

[jpm13095-bib-0012] Castelnuovo, G. , Gaggioli, A. , Mantovani, F. , & Riva, G. (2003). From psychotherapy to e‐therapy: The integration of traditional techniques and new communication tools in clinical settings. Cyberpsychology & Behavior: The Impact of the Internet, Multimedia and Virtual Reality on Behavior and Society, 6, 375–382. 10.1089/109493103322278754 14511449

[jpm13095-bib-0013] Chewning, B. , Bylund, C. L. , Shah, B. , Arora, N. K. , Gueguen, J. A. , & Makoul, G. (2012). Patient preferences for shared decisions: A systematic review. Patient Education and Counseling, 86(1), 9–18. 10.1016/j.pec.2011.02.004 21474265 PMC4530615

[jpm13095-bib-0014] Chong, W. W. , Aslani, P. , & Chen, T. F. (2011). Effectiveness of interventions to improve antidepressant medication adherence: A systematic review: Effectiveness of interventions to improve antidepressant adherence. International Journal of Clinical Practice, 65(9), 954–975. 10.1111/j.1742-1241.2011.02746.x 21849010

[jpm13095-bib-0015] Clever, S. L. , Ford, D. E. , Rubenstein, L. V. , Rost, K. M. , Meredith, L. S. , Sherbourne, C. D. , Wang, N.‐Y. , Arbelaez, J. J. , & Cooper, L. A. (2006). Primary care patients' involvement in decision‐making is associated with improvement in depression. Medical Care, 44(5), 398–405. 10.1097/01.mlr.0000208117.15531.da 16641657

[jpm13095-bib-0016] Confederación Española de Agrupaciones de Familiares y Personas con Enfermedad Mental . (2014). Guía Partisam: Promoción de la participación y autonomía en salud mental . Asociación Española de Neuropsiquiatría.

[jpm13095-bib-0017] Crawford, M. J. , Rutter, D. , Manley, C. , Weaver, T. , Bhui, K. , Fulop, N. , & Tyrer, P. (2002). Systematic review of involving patients in the planning and development of health care. British Medical Journal, 325(7375), 1263. 10.1136/bmj.325.7375.1263 12458240 PMC136920

[jpm13095-bib-0018] Davidson, L. , & Roe, D. (2007). Recovery from versus recovery in serious mental illness: One strategy for lessening confusion plaguing recovery. Journal of Mental Health, 16(4), 459–470. 10.1080/09638230701482394

[jpm13095-bib-0019] De Las Cuevas, C. , & Peñate, W. (2016). Validity of the control preferences scale in patients with emotional disorders. Patient Preference and Adherence, 10, 2351–2356. 10.2147/PPA.S122377 27895470 PMC5118017

[jpm13095-bib-0020] Department of Health . (2010). Liberating the NHS. The Stationary Office Limited.

[jpm13095-bib-0021] Department of Health . (2012). Liberating the NHS: No decision about me, without me. The Stationary Office Limited.

[jpm13095-bib-0080] Diaz del Peral, D. (Coord.) (2011). Ansiedad, depresión, somatizaciones: proceso asistencial integrado (2nd ed.). Consejeria de Salud, Junta de Andalucia.

[jpm13095-bib-0022] Drake, R. E. , Deegan, P. E. , & Rapp, C. (2010). The promise of shared decision making in mental health. Psychiatric Rehabilitation Journal, 34(1), 7–13. 10.2975/34.1.2010.7.13 20615839

[jpm13095-bib-0023] Duncan, E. , Best, C. , & Hagen, S. (2010). Shared decision making interventions for people with mental health conditions. Cochrane Database of Systematic Reviews, 2010, CD007297. 10.1002/14651858.CD007297.pub2 20091628 PMC7209977

[jpm13095-bib-0024] Edwards, N. , Walker, S. , Paddick, S.‐M. , Prina, A. M. , Chinnasamy, M. , Reddy, N. , Mboya, I. B. , Mtei, M. , Varghese, M. , Nakkasuja, N. , Guerra, M. , Sapkota, N. , & Dotchin, C. (2023). Prevalence of depression and anxiety in older people in low‐ and middle‐ income countries in Africa, Asia and South America: A systematic review and meta‐analysis. Journal of Affective Disorders, 325, 656–674. 10.1016/j.jad.2023.01.068 36681304

[jpm13095-bib-0025] Flückiger, C. , Forrer, L. , Schnider, B. , Bättig, I. , Bodenmann, G. , & Zinbarg, R. E. (2016). A single‐blinded, randomized clinical trial of how to implement an evidence‐based treatment for generalized anxiety disorder [IMPLEMENT]—Effects of three different strategies of implementation. eBioMedicine, 3, 163–171. 10.1016/j.ebiom.2015.11.049 26870827 PMC4739431

[jpm13095-bib-0026] Foa, E. B. , Liebowitz, M. R. , Kozak, M. J. , Davies, S. , Campeas, R. , Franklin, M. E. , Huppert, J. D. , Kjernisted, K. , Rowan, V. , Schmidt, A. B. , Simpson, H. B. , & Tu, X. (2005). Randomized, placebo‐controlled trial of exposure and ritual prevention, clomipramine, and their combination in the treatment of obsessive‐compulsive disorder. The American Journal of Psychiatry, 162(1), 151–161. 10.1176/appi.ajp.162.1.151 15625214

[jpm13095-bib-0027] García‐Herrera Pérez‐Bryan, J. M. , Hurtado Lara, M. M. , Nogueras Morilla, V. E. , Bordallo Aragón, A. , & Morales Asensio, J. M. (2015). Guía sobre el trastorno de ansiedad generalizada (TAG) en versión rápida y completa. UGC Salud Mental Hospital Regional Málaga.

[jpm13095-bib-0028] García‐Herrera Pérez‐Bryan, J. M. , Nogueras, V. E. , Muñoz Cobos, F. , & Morales Asensio, J. M. (2011). Guía de Práctica Clínica para el tratamiento de la depresión en Atención Primaria . http://www.sspa.juntadeandalucia.es/servicioandaluzdesalud/publicaciones/listadodeterminado.asp?idp=568

[jpm13095-bib-0029] Geramita, E. M. , Herbeck Belnap, B. , Abebe, K. Z. , Rothenberger, S. D. , Rotondi, A. J. , & Rollman, B. L. (2018). The association between increased levels of patient engagement with an internet support group and improved mental health outcomes at 6‐month follow‐up: Post‐hoc analyses from a randomized controlled trial. Journal of Medical Internet Research, 20(7), e10402. 10.2196/10402 30021711 PMC6068384

[jpm13095-bib-0030] Goossensen, A. , Zijlstra, P. , & Koopmanschap, M. (2007). Measuring shared decision making processes in psychiatry: Skills versus patient satisfaction. Patient Education and Counseling, 67, 50–56. 10.1016/j.pec.2007.01.017 17350214

[jpm13095-bib-0031] Goss, C. , Moretti, F. , Mazzi, M. A. , Del Piccolo, L. , Rimondini, M. , & Zimmermann, C. (2008). Involving patients in decisions during psychiatric consultations. The British Journal of Psychiatry: the Journal of Mental Science, 193(5), 416–421. 10.1192/bjp.bp.107.048728 18978325

[jpm13095-bib-0032] Griffiths, K. M. , Mackinnon, A. J. , Crisp, D. A. , Christensen, H. , Bennett, K. , & Farrer, L. (2012). The effectiveness of an online support group for members of the community with depression: A randomised controlled trial. PLoS One, 7(12), e53244. 10.1371/journal.pone.0053244 23285271 PMC3532446

[jpm13095-bib-0033] Guba, E. , & Lincoln, Y. (2000). Paradigmatic controversies, contradictions, and emerging confluences. In Handbook of qualitative research (2nd ed., pp. 163–188). Sage Publications.

[jpm13095-bib-0034] Gurtner, C. , Schols, J. M. G. A. , Lohrmann, C. , Halfens, R. J. G. , & Hahn, S. (2021). Conceptual understanding and applicability of shared decision‐making in psychiatric care: An integrative review. Journal of Psychiatric and Mental Health Nursing, 28(4), 531–548. 10.1111/jpm.12712 33191536

[jpm13095-bib-0035] Haug, T. , Nordgreen, T. , Öst, L.‐G. , Tangen, T. , Kvale, G. , Hovland, O. J. , Heiervang, E. R. , & Havik, O. E. (2016). Working alliance and competence as predictors of outcome in cognitive behavioral therapy for social anxiety and panic disorder in adults. Behaviour Research and Therapy, 77, 40–51. 10.1016/j.brat.2015.12.004 26708332

[jpm13095-bib-0036] Hodges, B. D. , Kuper, A. , & Reevez, S. (2008). Qualitative research: Discourse analysis. British Medical Journal, 337(7669), 570–572. 10.1136/bmj.a879

[jpm13095-bib-0037] Hoyer, J. , Beesdo, K. , Gloster, A. , Runge, J. , Höfler, M. , & Becker, E. (2009). Worry exposure versus applied relaxation in the treatment of generalized anxiety disorder. Psychotherapy and Psychosomatics, 78, 106–115. 10.1159/000201936 19218829

[jpm13095-bib-0038] Hurtado, M. M. , Macías, M. , & Morales‐Asencio, J. M. (2023). A new form of checking obsessive‐compulsive disorder in physicians: Another consequence of the COVID‐19 pandemic. A case series. Psychiatry Research Case Reports, 2(1), 100085. 10.1016/j.psycr.2022.100085 36533208 PMC9749375

[jpm13095-bib-0039] Johnson, S. , Price, M. , Mehta, N. , & Anderson, P. (2014). Stereotype confirmation concerns predict dropout from cognitive behavioral therapy for social anxiety disorder. BMC Psychiatry, 14, 233. 10.1186/s12888-014-0233-8 25199046 PMC4149193

[jpm13095-bib-0040] Joosten, E. A. , DeFuentes‐Merillas, L. , de Weert, G. H. , Sensky, T. , van der Staak, C. P. , & de Jong, C. A. (2008). Systematic review of the effects of shared decision‐making on patient satisfaction, treatment adherence and health status. Psychotherapy and Psychosomatics, 77(4), 219–226. 10.1159/000126073 18418028

[jpm13095-bib-0041] Levy Berg, A. , Sandahl, C. , & Clinton, D. (2008). The relationship of treatment preferences and experiences to outcome in generalized anxiety disorder (GAD). Psychology and Psychotherapy: Theory, Research and Practice, 81(3), 247–259. 10.1348/147608308X297113 18435869

[jpm13095-bib-0042] Liebherz, S. , Härter, M. , Dirmaier, J. , & Tlach, L. (2015). Information and decision‐making needs among people with anxiety disorders: Results of an online survey. The Patient–Patient‐Centered Outcomes Research, 8(6), 531–539. 10.1007/s40271-015-0116-1 25663124

[jpm13095-bib-0043] Loh, A. , Simon, D. , Wills, C. E. , Kriston, L. , Niebling, W. , & Härter, M. (2007). The effects of a shared decision‐making intervention in primary care of depression: A cluster‐randomized controlled trial. Patient Education and Counseling, 67(3), 324–332. 10.1016/j.pec.2007.03.023 17509808

[jpm13095-bib-0044] Marshall, T. , Stellick, C. , Abba‐Aji, A. , Lewanczuk, R. , Li, X.‐M. , Olson, K. , & Vohra, S. (2021). The impact of shared decision‐making on the treatment of anxiety and depressive disorders: Systematic review. BJPsych Open, 7(6), e189. 10.1192/bjo.2021.1028 PMC8612021 34784989

[jpm13095-bib-0045] Matthias, M. S. , Salyers, M. P. , Rollins, A. L. , & Frankel, R. M. (2012). Decision making in recovery‐oriented mental health care. Psychiatric Rehabilitation Journal, 35(4), 305–314. 10.2975/35.4.2012.305.314 22491370 PMC3980461

[jpm13095-bib-0046] Mavranezouli, I. , Mayo‐Wilson, E. , Dias, S. , Kew, K. , Clark, D. M. , Ades, A. E. , & Pilling, S. (2015). The cost effectiveness of psychological and pharmacological interventions for social anxiety disorder: A model‐based economic analysis. PLoS One, 10(10), e0140704. 10.1371/journal.pone.0140704 26506554 PMC4624770

[jpm13095-bib-0047] McCabe, R. , Khanom, H. , Bailey, P. , & Priebe, S. (2013). Shared decision‐making in ongoing outpatient psychiatric treatment. Patient Education and Counseling, 91(3), 326–328. 10.1016/j.pec.2012.12.020 23414657

[jpm13095-bib-0048] McCormack, B. , Karlsson, B. , Dewing, J. , & Lerdal, A. (2010). Exploring person‐centredness: A qualitative meta‐synthesis of four studies: Person‐centredness: A qualitative meta‐synthesis. Scandinavian Journal of Caring Sciences, 24(3), 620–634. 10.1111/j.1471-6712.2010.00814.x 21050249

[jpm13095-bib-0049] McCormack, B. , & McCance, T. (2016). Person‐Centred practice in nursing and health care: Theory and practice (2nd ed.). Wiley.

[jpm13095-bib-0050] Menear, M. , Dugas, M. , Careau, E. , Chouinard, M.‐C. , Dogba, M. J. , Gagnon, M.‐P. , Gervais, M. , Gilbert, M. , Houle, J. , Kates, N. , Knowles, S. , Martin, N. , Nease, D. E. , Zomahoun, H. T. V. , & Légaré, F. (2020). Strategies for engaging patients and families in collaborative care programs for depression and anxiety disorders: A systematic review. Journal of Affective Disorders, 263, 528–539. 10.1016/j.jad.2019.11.008 31744737

[jpm13095-bib-0051] Menear, M. , Girard, A. , Dugas, M. , Gervais, M. , Gilbert, M. , & Gagnon, M.‐P. (2022). Personalized care planning and shared decision making in collaborative care programs for depression and anxiety disorders: A systematic review. PLoS One, 17(6), e0268649. 10.1371/journal.pone.0268649 35687610 PMC9187074

[jpm13095-bib-0052] Mohr, D. C. , Burns, M. N. , Schueller, S. M. , Clarke, G. , & Klinkman, M. (2013). Behavioral intervention technologies: Evidence review and recommendations for future research in mental health. General Hospital Psychiatry, 35(4), 332–338. 10.1016/j.genhosppsych.2013.03.008 23664503 PMC3719158

[jpm13095-bib-0053] Mundal, I. , Lara‐Cabrera, M. L. , Betancort, M. , & De Las Cuevas, C. (2021). Exploring patterns in psychiatric outpatients' preferences for involvement in decision‐making: A latent class analysis approach. BMC Psychiatry, 21(1), 133. 10.1186/s12888-021-03137-x 33676452 PMC7937224

[jpm13095-bib-0054] National Collaborating Centre for Mental Health . (2006). Obsessive‐compulsive disorder: Core interventions in the treatment of obsessive‐compulsive disorder and body dysmorphic disorder . British Psychological Society. 21834191

[jpm13095-bib-0055] National Collaborating Centre for Mental Health . (2013). Social anxiety disorder: Recognition assessment and treatment. British Psychological Society. 25577940

[jpm13095-bib-0056] National Institute for Health and Care Excellence: Clinical Guidelines . (2011). Generalised anxiety disorder and panic disorder (with or without agoraphobia) in adults management in primary, secondary and community care clinical practice guidelines. British Psychological Society.

[jpm13095-bib-0057] Onocko‐Campos, R. T. , Díaz, A. R. G. , Dahl, C. M. , Leal, E. M. , & Serpa Junior, O. D. D. (2017). Methodological challenges in the use of focus groups with people with severe mental illness. Cadernos de Saúde Pública, 33(6), e00187316. 10.1590/0102-311x00187316 28724031

[jpm13095-bib-0058] Patel, S. R. , & Bakken, S. (2010). Preferences for participation in decision making among ethnically diverse patients with anxiety and depression. Community Mental Health Journal, 46(5), 466–473. 10.1007/s10597-010-9323-3 20556512 PMC2931338

[jpm13095-bib-0059] Ramos‐García, V. , Rivero‐Santana, A. , Duarte‐Díaz, A. , Perestelo‐Pérez, L. , Peñate‐Castro, W. , Álvarez‐Pérez, Y. , González‐González, A. I. , & Serrano‐Aguilar, P. (2021). Shared decision‐making and information needs among people with generalized anxiety disorder. European Journal of Investigation in Health, Psychology and Education, 11(2), 423–435. 10.3390/ejihpe11020031 34708821 PMC8314357

[jpm13095-bib-0060] Rapee, R. M. , Gaston, J. E. , & Abbott, M. J. (2009). Testing the efficacy of theoretically derived improvements in the treatment of social phobia. Journal of Consulting and Clinical Psychology, 77(2), 317–327. 10.1037/a0014800 19309190

[jpm13095-bib-0061] Remes, O. , Brayne, C. , Van Der Linde, R. , & Lafortune, L. (2016). A systematic review of reviews on the prevalence of anxiety disorders in adult populations. Brain and Behavior, 6(7), e00497. 10.1002/brb3.497 27458547 PMC4951626

[jpm13095-bib-0062] Rodenburg‐Vandenbussche, S. , Carlier, I. , Vliet, I. , Hemert, A. , Stiggelbout, A. , & Zitman, F. (2020). Patients' and clinicians' perspectives on shared decision‐making regarding treatment decisions for depression, anxiety disorders, and obsessive‐compulsive disorder in specialized psychiatric care. Journal of Evaluation in Clinical Practice, 26(2), 645–658. 10.1111/jep.13285 31612578

[jpm13095-bib-0063] Rollman, B. L. , Herbeck Belnap, B. , Abebe, K. Z. , Spring, M. B. , Rotondi, A. J. , Rothenberger, S. D. , & Karp, J. F. (2018). Effectiveness of online collaborative Care for Treating Mood and Anxiety Disorders in primary care: A randomized clinical trial. JAMA Psychiatry, 75(1), 56–64. 10.1001/jamapsychiatry.2017.3379 29117275 PMC5833533

[jpm13095-bib-0064] Schladitz, K. , Weitzel, E. C. , Löbner, M. , Soltmann, B. , Jessen, F. , Pfennig, A. , Riedel‐Heller, S. G. , & Gühne, U. (2023). Experiencing (shared) decision making: Results from a qualitative study of people with mental illness and their family members. Healthcare, 11(16), 2237. 10.3390/healthcare11162237 37628436 PMC10454232

[jpm13095-bib-0065] Shepherd, G. , Boardman, J. , & Slade, M. (2008). Making recovery a reality . Sainsbury Centre for Mental Health. https://www.centreformentalhealth.org.uk/sites/default/files/2018‐09/Making%20recovery%20a%20reality%20policy%20paper.pdf

[jpm13095-bib-0066] Simmons, M. B. , Hetrick, S. E. , & Jorm, A. F. (2011). Experiences of treatment decision making for young people diagnosed with depressive disorders: A qualitative study in primary care and specialist mental health settings. BMC Psychiatry, 11(1), 194. 10.1186/1471-244X-11-194 22151735 PMC3266645

[jpm13095-bib-0067] Song, M. , & Song, Y.‐M. (2023). The effect of shared decision‐making by mental health nurses on medication adherence in patients with alcohol use disorders: Provider‐patient communication pathway model. Journal of Health Communication, 28(11), 777–788. 10.1080/10810730.2023.2268561 37823392

[jpm13095-bib-0068] Storm, M. , & Edwards, A. (2013). Models of user involvement in mental health. In M. A. Keating , A. M. McDermott , & K. Montgomery (Eds.), Patient‐Centred health care: Achieving coordination, communication and innovation (pp. 214–277). Palgrave Macmillan. 10.1057/9781137308931_17

[jpm13095-bib-0069] Tambuyzer, E. , & Van Audenhove, C. (2015). Is perceived patient involvement in mental health care associated with satisfaction and empowerment? Health Expectations, 18(4), 516–526. 10.1111/hex.12052 23425015 PMC5060796

[jpm13095-bib-0070] Taylor, S. J. , Bogdan, R. , & DeVault, M. (2015). Introduction to qualitative research methods: A guidebook and resource (4th ed.). Wiley.

[jpm13095-bib-0071] Tlach, L. , Wüsten, C. , Daubmann, A. , Liebherz, S. , Härter, M. , & Dirmaier, J. (2015). Information and decision‐making needs among people with mental disorders: A systematic review of the literature. Health Expectations, 18(6), 1856–1872. 10.1111/hex.12251 25145796 PMC5810695

[jpm13095-bib-0072] Tolin, D. F. , Diefenbach, G. J. , & Gilliam, C. M. (2011). Stepped care versus standard cognitive‐behavioral therapy for obsessive‐compulsive disorder: A preliminary study of efficacy and costs. Depression and Anxiety, 28(4), 314–323. 10.1002/da.20804 21381157 PMC3480726

[jpm13095-bib-0073] Val, A. , & Míguez, M. C. (2023). Prevalence of antenatal anxiety in European women: A literature review. International Journal of Environmental Research and Public Health, 20(2), 1098. 10.3390/ijerph20021098 36673854 PMC9858852

[jpm13095-bib-0074] van Apeldoorn, F. J. , Stant, A. D. , van Hout, W. J. , Mersch, P. P. , & den Boer, J. A. (2014). Cost‐effectiveness of CBT, SSRI, and CBT+SSRI in the treatment for panic disorder. Acta Psychiatrica Scandinavica, 129(4), 286–295. 10.1111/acps.12169 23834587

[jpm13095-bib-0075] Vergouwen, A. C. M. , Baker, A. , Katon, W. J. , Verheji, T. J. , & Koerselmar, F. (2003). Improving adherence to antidepressants: A systematic review of interventions. Journal of Clinical Psychiatry, 64(12), 1415–1420. 14728101 10.4088/jcp.v64n1203

[jpm13095-bib-0076] Vinogradov, S. , & Yalom, I. D. (1996). Guía breve de psicoterapia de grupo. Paidós.

[jpm13095-bib-0077] WHO . (2022). International Statistical Classification of Diseases and Related Health Problems . https://www.who.int/standards/classifications/classification‐of‐diseases

[jpm13095-bib-0078] Zabinski, M. F. , Celio, A. A. , Wilfley, D. E. , & Taylor, C. B. (2003). Prevention of eating disorders and obesity via the internet. Cognitive Behaviour Therapy, 32(3), 137–150. 10.1080/16506070310000939 16291545

[jpm13095-bib-0079] Zhang, S. X. , Chen, R. Z. , Xu, W. , Yin, A. , Dong, R. K. , Chen, B. Z. , Delios, A. Y. , Miller, S. , McIntyre, R. S. , Ye, W. , & Wan, X. (2022). A systematic review and meta‐analysis of symptoms of anxiety, depression, and insomnia in Spain in the COVID‐19 crisis. International Journal of Environmental Research and Public Health, 19(2), 1018. 10.3390/ijerph19021018 35055841 PMC8775436

